# An Essay on Medical Philosophy, and on the Generalities of Clinical Medicine, &c.

**Published:** 1837-04

**Authors:** 


### Art. VI.

*Essai sur la Philosophie Médicale, et sur les Généralités de la Clinique Médicale; précédé d'un Résumé Philosophique des principaux Progrès de la Médecine, et suivi d'un Parallèle des Résultats de la Formule des Saignées coup sur coup avec ceux de l'ancienne Méthode, dans le Traitement des Phlegmasies Aigues.* Par J. Bouillaud, Professeur de Clinique Médicale à la Faculté de Médecine de Paris.—*Paris,* 1836. 8vo. pp. 426.

*An Essay on Medical Philosophy, and on the Generalities of Clinical Medicine, &c.* By J. Bouillaud, &c.

The professed intention of M. Bouillaud in this work is to impart to the study of medicine the exactness of physical science; an attempt which he acknowledges not to be new, but which he conceives himself enabled to make with more success than his predecessors. Of the desirableness of reducing the practice of medicine to fixed and invariable principles, no doubt can be entertained; but a question may very fairly be entertained as to the sufficient advancement of medicine for such an attempt at present; and we believe the general conviction of reflecting men to be so decidedly unfavorable to a design so ambitious, as to make the perusal of works professing to maintain it somewhat distasteful to them. We must acknowledge such to be our own case; and, although we admire the extensive views and eloquent declamation of M. Bouillaud, and can conceive his subject expanded over twenty volumes instead of one, we fear we should close the twentieth as we close the first, without being converted to his belief.

M. Bouillaud's work is divided into four parts, which might have formed four distinct essays; the first appearing to be a kind of history of medicine; the second, a sort of clinical guide; the third relates to semeiology, and the theory of medicine; and the fourth is a mere comparison between recent and ancient methods of curing acute inflammations.

The most interesting portion of the historical division of the work is, as might be expected, that which relates to the cultivation of medicine by some of the modern French physiologists and pathologists. This is prefaced by a very discursive review of former systems, ancient and modern; which we shall pass over, and come at once to the epoch of Bichat and Pinel. These two great reformers of medicine appeared immediately after that great political revolution which it was prognosticated would overthrow the arts and sciences. Of the opinions of these celebrated men M. Bouillaud gives a clear and animated account, not without great interest to the medical philosopher or student.

To Bichat, he says, belongs the glory of having conceived and executed the plan of a new anatomy, that of certain immediate elements of composite organs, to which he gave the name of *General Anatomy*, or the anatomy of general systems, or of systems the generators of organs, (*des systèmes généraux, ou générateurs des organes.*) He professed to be a follower of Stahl, whose doctrine he modified with the power of an original genius. He departed from him in not ascribing all the vital phenomena to a single principle, that principle to which Van Helmont had given the name of *Archæus,* and which Barthez had named the *vital principle,* and Chaussier the *vital force.* This principle Bichat regarded as purely an imaginary abstraction; whilst the object of his own doctrine was, to analyze with precision the properties of living bodies; to shew that every physiological phenomenon was ultimately related to these properties, considered in their natural condition, and every pathological phenomenon derived from their augmentation, diminution, or alteration; that every therapeutical phenomenon had for its principle the return of these properties to their natural type; and to fix with precision the circumstances in which each was called into action; to distinguish, in physiology as in medicine, what proceeded from each, and consequently to determine exactly the natural and morbid phenomena governed by the animal or produced by the organic functions; to indicate when the animal sensibility and contractility, and when the organic sensibility, and sensible or insensible contractilities, were concerned. He repudiated the application of the laws of the physical sciences to explain vital phenomena. Discarding the affinities of chemistry, and the elasticity and gravity of physics, he employed in physiology the terms sensibility and contractility; always making an exception in favour of those cases in which, as with regard to the eye or ear, the same organ was the seat of vital and of physical phenomena.

According to the views of Bichat, then, sensibility and contractility are the causes of all the phenomena observed in organized bodies; and all diseases are but lesions of these properties, and are numerous in proportion to the number of vital properties with which organized bodies are endowed. He considered nervous disorders to arise from lesions of animal sensibility; and convulsions and paralysis to consist of an increase or diminution of animal contractility. In all the class of fevers, and all gastric affections, he looked upon disturbance of the organic contractility as manifest; and he ascribed tumours, marasmus, and increased exhalation, to disturbance of the organic sensibility, and in the insensible contractility corresponding to it.

Bichat's system involved him in an evident difficulty. He considered the seat of the vital properties to be the solids; and, as the phenomena of disease were but alterations of the vital properties, his views seemed to exclude the fluids from consideration, as regarded the phenomena of disease. Yet he was far from overlooking the importance of the fluids, and knew that, although their influence had been over-rated, they were often at least the vehicle of morbific matter, especially such of them as were destined to the composition of the organs; and that, although in all diseases the solids were most palpably affected, the cause of the affection was not invariably in themselves, he concluded that a pathological doctrine, based upon exclusive solidism or fluidism, was as unreasonable as a physiological theory admitting only the solids, or only the fluids.

To adapt to these views his doctrine of vital properties, M. Bouillaud seems to think that it was necessary to admit the vitality of the fluids themselves, which Bichat certainly did, although the philosophical reasonableness of that opinion was doubtless sufficient to recommend it, independently of all views of convenience.

Bichat applied his doctrines respecting the organized elements of the body, and the simple tissues, and general systems, to the analogies and distinctions of maladies; especially local maladies, acute or chronic. He especially excepted the greater number of fevers, which, simultaneously affecting all parts, he did not consider capable of having much light thrown upon them by the anatomy of systems. Against this exception, however, M. Bouillaud, an ardent follower of Broussais, lifts up his voice in a succession of foot-notes, asserting, of course, the localization of fevers themselves. Since all maladies, reasoned Bichat, are but alterations of vital properties, and each tissue differs from others in respect to these properties, it is evident that it must also differ in respect to its maladies; and in every organ composed of different tissues, one may be diseased, and the rest intact. As every organized tissue has a uniform disposition, and, whatever its situation, is identical in structure and properties, its diseases must be everywhere the same. Whether the serous tissue forms the arachnoid, the pleura, the pericardium, or the peritoneum, it becomes inflamed in the same manner, dropsical effusions follow, whitish miliary tubercles are formed, &c. He applied the same observations to the mucous tissues; and he extended the application from the history of diseases to their consequences, as shewn by morbid anatomy. In accordance with his general views, he maintained that no medicament, not even a poultice, acted physically on the frame, but solely by modifying the vital properties. His therapeutics, although evidently too exclusive in principle, seemed indeed to derive recommendation from the errors to which he opposed them. He pointed out with the force of truth the erroneous employment and denomination of many classes of medicines; such as the term deobstruants, which originated in the theory of obstructions; incisives, which were directed against a presumed thickening of the humours; astringents and relaxants, which belonged to the doctrine of *strictum* and *laxum;* and refrigerants and calorifiants, which regarded the excess or defect of animal heat. But, although he pointed out the weakness of theories of this kind, he was scarcely less at a loss than other reasoners to devise a classification of medicines founded on their real mode of action; although he enounced, as a general principle, that all medicines had for their object to bring back the vital forces to the natural type from which they had departed in disease. Thus, in inflammations, he says, as there is exaltation of the organic sensibility and insensible contractility, we must diminish this exaltation by poultices, fomentations, baths, &c.

Amidst the truths contained in these theories of Bichat, and which were received with enthusiasm, and hailed as brilliant discoveries founded on the ruins of what the reflecting reader may perhaps consider to have been nearly the same theory in other forms, it is not difficult to perceive great defects. The doctrine of vitalism might be more intelligible, or less open to perversion, than that of a *vis insita*, an *impetum faciens*, an *archeus*, a *vital force;* but, as M. Bouillaud truly remarks, the terms organic sensibility, and organic contractility, sensible and insensible, are deficient in clearness, and the impropriety of excluding physical laws from consideration in reasoning on the functions of the human body will now be universally admitted. The name of Bichat will, however, be imperishably remembered in connexion with his Treatise on the Membranes, his General Anatomy, and his Researches on Life and Death; works which exhibit all the characteristics of an original and philosophical mind. To his labours, and to his example, M. Bouillaud ascribes much of the honour of having formed a Dupuytren (whom he rather extravagantly calls the *Napoleon* of surgery), a Cruveilhier, a Richerand, and lastly, Broussais.

The other great name on which M. Bouillaud dwells with just admiration is that of Pinel. This accomplished physician proceeded to the task of reforming and arranging nosological medicine, prepared by an ample acquaintance with its history and actual advancement. During the many years in which his popular *Nosographie Philosophique* was undergoing several successive editions, the gradual improvements effected by different cultivators of the science did not escape his attention; and it ensued that he both felt and acknowledged the increasing difficulties in the way of making a satisfactory arrangement of diseases. Of his five great classes, Fevers, Inflammations, Hemorrhages, Neuroses, and Organic Lesions, the first was surrounded with new difficulties every time that he brought his consideration to the subject, and the last comprehended affections exhibiting a disparity almost defying all attempts to effect a regular distribution of them. The researches of Rœderer and Wagler concerning mucous fever (la fièvre muqueuse), those of Sarcone on the glutinous fever, and his own, concerning gastric and meningeal fevers, tended to shake the foundations of his first class altogether; and he is supposed, even in his earliest publications, to have had a tendency to localize fevers, or to deny the essentiality which the modern French school so utterly and vehemently rejects. He states his principal design to have been to present purely historical descriptions of the entire course of maladies, and abstract notions of general pathology, which he considered to constitute medical science. Pitcairn, one of the mathematical sect of physicians, had enunciated a problem in these terms,—a disease being given, to find the remedy; and in the place of this somewhat presumptuous question, Pinel wished to substitute what he considered a problem more circumscribed and reasonable,—a malady being given, to determine its real character, and its nosological place. The modern reader will feel inclined to condemn both these problems; the first as savouring of scholastic trifling, the second as placing too far back the first object of all medicine, the curing of diseases.

In the mean time, the great subject of fever was continually undergoing further elucidation, and the observations of Prost, in a work entitled *Médecine éclairée par l'ouverture des corps*, and the celebrated monograph of Petit and Serres on *Entero-mesenteric* fever, are alluded to by M. Bouillaud as having particularly tended to overthrow Class I. of the Nosographie; whilst Class V., or that of Organic Lesions, sustained as severe an assault from M. Broussais's famous publication on Chronic Inflammations. Between this distinguished pathologist and Pinel there ensued a lively war of opinion. Of this, and of the opinions of Prost, of Laennec, of Petit and Serres, and others who materially contributed to correct those views of fever which overlooked local lesions, more especially of the intestinal canal, the reader will find a very interesting account in the sections of M. Bouillaud's work entitled *Décadence du Système de Pinel*, and a subsequent one entitled *Ecole de M. Broussais.*

The doctrines of Broussais have undergone such abundant discussion during the last fifteen years, in the journals of this country, as to render it unnecessary to lay before our readers any long account of them. Qualified for the task by habits of observation and boldness of character, he found the demolition of the artificial nosologies of the time no very difficult undertaking; and it was his ambition and his boast to establish a system of medicine not founded on the arbitrary classification of symptoms, but on anatomy and physiology. He maintained that the symptoms should be referred to the organs of which they indicated the disorders, and that it should also be enquired why and how the organ was affected, and in what manner it was possible to relieve the affection. One of his leading propositions related to the subversion of the essentiality of fevers, and their localization in the digestive canal, of which they were affirmed to be an acute inflammation, seated in the mucous or follicular membrane. The universality of this occurrence has been repeatedly disproved; but the observation of it was important, and even the undue, because too general, weight attached to it by M. Broussais tended to effect material improvement in the management of fevers by the continental practitioners. For the next great division of his attack upon established systems he selected M. Pinel's fifth class, or that of Organic Lesions, which he pronounced to be a *chef d'œuvre* of contradictions and inconsistencies, in which syphilis, gangrene, scorbutus, cancer, dropsies, diabetes, worms, &c. were ranged together: he argued that syphilis was not more truly disorganizing than phlegmon, catarrh, and dysentery; that cancer and tubercles were connected with chronic inflammations; and that, in short, the term organic lesions, like the older term of cachexies, was but calculated to flatter the ignorance of physicians, and prevent their ascending to the true causes of such affections. Although he admitted that sometimes degenerations and transformations were insensibly effected without any marked preceding pathological state, he considered that the mechanism of these obscurely-wrought changes might be illustrated by those which were produced in a manner more evident. He ascribed, we have said, generally, tuberculous and cancerous degenerations to an inflammatory process, not analogous to that of phlegmon, but existing in the white vessels, or a lymphatic inflammation, which he subsequently denominated an irritation; and he urged the study of *irritations* in the different tissues, in order to the comprehension of a state distinct from phlegmon, and yet not asthenic, spasmodic, or engorged, terms which he denounced. This idea of irritations he acknowledged to have deduced from Bichat, and he expressed himself in no measured terms concerning those who did not comprehend it. He also denied the dependence of scorbutus on mere debility, referring it to an alteration or corruption of the blood.

Perhaps, on concluding the perusal of M. Bouillaud's account of his great master's opinions, and their triumph over opposition, the reader may be not a little inclined to wonder at the small number of new ideas which seem required, on which to found the reputation of a reformer of medicine; and, with us, to look back upon the fierce controversies which attended the downfal of *Pinelism* and the uprising of *Broussaisism*, as too much resembling the verbose disputes which preserve the life of medical debating societies. Yet never were partisans more zealous, never were opponents more bitter, than those created by M. Broussais's physiological doctrine. Some of his pupils, filled with one idea, saw irritation everywhere, whilst others overlooked it where it existed. The practical consequences were numerous, and not always of a fortunate character. Every unprejudiced observer of Parisian practice must have seen the evil consequences of the excessive application of leeches to the abdomen, conjoined with too strict a diet. The least tenderness of the epigastrium under rough pressure has often been held an imperative reason for applying numerous leeches; and, the superficial tenderness being found to be increased the next day, their repetition has followed again and again, whilst gum-water and starvation did the rest. In vain the pallid face, the prominent malar bones, the imploring look, the earnest prayer for food. Much complacency was indulged in the contemplation of the march of maladies, and if death did not follow, the patient was regarded as one who had defrauded the zealous pupils of a dissection. But the patient seldom escaped with life, and beds were emptied, especially in the children's hospital, with a frightful rapidity. We speak of what we have seen, and the mortality of the wards at the time to which we refer afforded horrible confirmation of these impressions. On the other hand, if no tenderness was found on pressure, and if the chest presented no morbid indication, the followers of Broussais, of whom they were many who denied the name, appeared incapable of recognizing a simple fever. It could not be a simple fever, for their master derided the notion of such essentialities: it was therefore, after a second exploration, set down as an imaginary malady. The unsteady step, the oppressed countenance, the loaded tongue, the quickened pulse, were disregarded. There was no thoracic or abdominal inflammation; nothing could be localized; it was an imagination. But fever held on its natural course, despite of theories untrue to nature, and in a few days the tongue grew darker, the brain was deeply disturbed, the hands trembled, and the Broussaian exclaimed, with an air of discovery, that the patient was affected with "fièvre grave," a sentence which death very soon confirmed. So true it is, that whatever madness affects the leaders of medicine, it is the patients who suffer. The exclusive theorist walks proudly round the wards, and behind him "mocking his state" is the figure death, who alone gains by the errors of human conceit in medicine. Such errors were generally committed by the most enthusiastic admirers of the great physiologist. Other errors were committed, as of old, by his opponents, who continued to treat debility in fevers as an entity, to be combated by tonics and stimulants. It was lamentable to perceive how few were free from the folly of seeing nothing but debility, or nothing but inflammation. Some few, however, there were, who endeavoured to fix upon the truth, which lay betwixt the contending parties, and they shared the abuse of both. The old school blamed their rashness, the Broussaians pitied their timidity; the old school lamented their proneness to innovation, the new deplored their attachment to exploded prejudices. The sagacious few were denominated the Eclectics; and their ranks appear to have gradually diminished. Among the converts to the doctrine of the non-essentiality of fevers under all circumstances, M. Bouillaud names Andral, Rostan, Lallemand, Boisseau, Roche, Bégin, Rayer, Dugès, Billard, Chauffard, and Scoutteten; whilst on the opposite side appear alone the names of Laennec and of Chomel, the latter, however, well known to be one of the best practitioners in France, and a physician of the calmest judgment and the most remarkable candour.

In a work published by M. Chomel in 1821, he condemned the unmeasured opinions of those who, observing an inflammatory state of the intestinal mucous membrane in numerous cases of fever, maintained that fever could not exist without it. The result of his own observation was, that in some cases there was no appreciable alteration found in the intestinal canal on examination after death in fevers; that in others there was only slight redness, very limited in extent; but that in the greatest number, that is to say in about three-fourths of the fatal cases, ulcers were found in the intestines, near the ileo-cæcal valve, and the corresponding mesenteric glands were red and tumefied; and he regarded these appearances as being very often the effect, and very rarely the cause, of the symptoms of fever. It is difficult to conceive an opinion more judiciously expressed; and although M. Bouillaud lays great stress on a more recent statement of M. Chomel, (in 1824,) that follicular inflammation of the intestines is so common in fevers that in five years he had not found an exception, we think this assertion may be admitted as regards the fatal cases, to which alone it refers, without committing M. Chomel to the doctrine of which M. Bouillaud is so anxious to see him a supporter. In truth, M. Chomel still maintains, that fever may exist *without* lesion of the intestinal follicles.

The controversial temperament of M. Broussais seems in some degree to have caused his opponents to withhold the meed of merit actually due to him. If not the discoverer, he at least saw and maintained the importance of the doctrine which taught the frequent implication of the intestinal canal in inflammation during fevers; but in his zeal to overthrow the belief of the essentiality of fevers, he carried the doctrine beyond the limits supported by general experience. Referring to the laws of life, he ascribed the greater number of diseases to irritation; but he expressly denies ascribing *all* diseases to that cause: his doctrine, he says, is *not* a doctrine of irritation, but a physiological doctrine, resting not on the exaltation of life, but on all the modifications of which life is susceptible, of which, however, he declares exaltation to be incomparably the most frequent. Whatever may occasionally have been his language, his declaration at the conclusion of his *examen des doctrines* is framed in a philosophical spirit: he does not pretend, he says, to offer a perfect work; he sees that others will go farther than he has done in determining the symptoms characteristic of lesions of each of the primitive tissues; and he declares that he shall see their efforts with the liveliest satisfaction. It cannot, indeed, be doubted, that his originality, energy, and active observation have exercised a powerful influence over the cultivators of all parts of medicine. We speak of the French cultivators more particularly, for they have left no part of the wide field of medicine untilled or unimproved. Exact observations have succeeded to vague generalities, and numerous organic lesions have been most usefully traced to the morbid actions in which they originate; and with these improvements, practical medicine has nearly, if not entirely, kept pace.

It was our intention to notice other portions of M. Bouillaud's work, but we are deterred from the task by the common-place matter which meets us in almost every page, and by the author's excessive diffuseness. It is difficult to imagine any description of readers who would derive pleasure from our dilating on the history of clinical institutions, or on

"the sciences in general, especially that of observation;" or on "the particular genius of medicine;" or on the "spirit or genius of invention, observation, and experimentation;" on the various methods of drawing up cases, or on the "theoretical, logical, and systematic spirit applied to facts in medicine;" or on the generalities of etiology and pathological anatomy, diagnosis, prognosis, and therapeutics; which subjects are extended over more than four hundred of the author's pages.

Altogether, we are compelled to say that this discursive work of M. Bouillaud might have remained in his portfolio without any detriment to his reputation. It is not easy to recognize in its various parts an accordance with its title and professed object. The author goes out of his way, especially in his numerous notes, to discuss extraneous matters, such as the Newtonian theory, phrenology, political influences, and other topics; evincing the fluency and vivacity of the writer rather than any higher qualities. We have mentioned his fixing upon M. Dupuytren, whose real character presented an union of the man of genius and the savage, the denomination of the Napoleon of surgery; but this is still exceeded by his proclaiming M. Broussais, who is also doubtless a man of genius, but the most furious of medical sectarians, the *Messiah* of Medicine. We cannot but consider these things as alien to that Medical Philosophy which M. Bouillaud professes such anxiety to establish.

It is, we are well aware, on the second part of his work that M. Bouillaud especially plumes himself. He repeatedly refers to it as containing his system of philosophy. To us it appears no more than a clever compendium of clinical directions, not remarkable for their novelty, and very perplexing by their number. Each section is, moreover, treated at such length that we fear the number of those who read the work through will be very small indeed. We cannot get rid of the suspicion that the book is made up of works composed at different times, and with different objects. The First Part is evidently an *Eloge* of Broussais; the Second, a Clinical Manual; the Third, a Treatise on Semeiology and Therapeutics; and the Fourth, a little Essay on Bloodletting. The medical philosophy of all this, we fairly confess, surpasses our comprehension. We can discern no stream of thought, no train of reasoning, no directness or singleness of purpose; and if we are permitted to draw a conclusion from the absence of all modern names and references, except of and to the author's countrymen, we should say that his information in no degree warranted even his professed undertaking.

